# Reliability and validity of the Korean version of the coronary revascularization outcome questionnaire

**DOI:** 10.1186/s12955-017-0615-y

**Published:** 2017-02-15

**Authors:** Minju Kim, JiYeong Seo, Jin-Yong Hwang, Ki Soo Park

**Affiliations:** 1 Department of Nursing, Dong-A University, 32 Daesingonwon-ro, Seo-Gu, Busan, 49201 Korea; 2 College of Nursing, Catholic University of Pusan, 57 Oryundae-ro, Geumjeong-gu, Busan, 46252 Korea; 3 Division of Cardiology, Department of Internal Medicine, Gyeongsang National University Hospital, Jinju, Korea; 4 Department of Internal Medicine and Institute of Health Sciences, Gyeongsang National University School of Medicine Jinju, 816-15 Jinjudaero, Jinju City, Gyeongnam 52727 Korea; 5 Department of Preventive Medicine and Institute of Health Sciences, Gyeongsang National University School of Medicine, 816-15 Jinjudaero, Jinju City, Gyeongnam 52727 Korea

**Keywords:** Coronary revascularization outcome questionnaire, Percutaneous coronary intervention, Quality of life, Reliability, Validity

## Abstract

**Background:**

People with ischemic heart disease have increased drastically, and their health-related quality of life (HRQOL) has been increasingly important. The Coronary Revascularization Outcome Questionnaire (CROQ) is a widely used tool to assess the quality of life in patients with coronary artery disease. The purpose of this study was to rigorously examine the psychometric properties of the CROQ in patients who had undergone percutaneous coronary intervention (PCI).

**Methods:**

The CROQ was translated into Korean. A total of 209 patients before PCI and 169 patients after PCI were recruited from a university hospital in Korea and completed questionnaires. In terms of statistical analyses, internal consistency, concurrent validity with using the Short Form 12 (SF-12) and Seattle Angina Questionnaire-Korean version (SAQ-K), and construct validity using exploratory factor analysis were assessed. Effective size statistics were calculated.

**Results:**

The internal consistency coefficients for all subscales of the CROQ were above 0.70, except the domain of adverse effects. The concurrent validity was mostly supported by the pattern of association among CROQ-K, SAQ-K, and SF-12. The results of EFA showed the core items of the CROQ had 7 factors. Large effect sizes were observed for the symptoms and the psychosocial functioning scales.

**Conclusions:**

The Korean version of the CROQ is a valid and reliable scale for assessing HRQOL in patients with coronary artery disease.

## Background

Ischemic heart disease (IHD) has been the leading cause of death worldwide [1]. In Korea, heart disease is the second major cause of death and has increased drastically due to the rapidly aging population and changes in diet [2]. The cause of death of 71,174 of the 267,692 people who died in 2014 was cardiovascular disease, including a considerable number of people who died due to ischemic heart disease [2]. With the high mortality associated with IHD, medical costs are significantly higher for this condition compared to other chronic diseases [3].

The development of new treatment methods and prevention programs has been studied to reduce the mortality and medical costs associated with IHD [4, 5]. Coronary revascularization, such as percutaneous coronary intervention (PCI) and coronary artery bypass graft surgery (CABG), is the most common procedure used to treat IHD [4, 6]. Many studies have evaluated the effectiveness of coronary revascularization by referring to the mortality rate or the results of clinical laboratory tests [4, 7]. However, mortality rates and laboratory findings do not fully reflect patient well-being [8]. Quality of life should also be considered to determine the optimum treatment.

The quality of life of patients with IHD rapidly declines due to uncertainty about the prognosis of the disease and the burden of medical expenses [9, 10]. In addition, there is a high risk of re-hospitalization due to the relapse of acute coronary syndrome or complications of the procedure [9, 11]. Therefore, disease-specific health-related quality of life (HRQOL) should be assessed to understand changes in quality of life in IHD patients after revascularization.

Some generic measures, such as the SF-36 and the Nottingham Health Profile (NHD), have been used to evaluate quality of life in patients after revascularization. However, these instruments were not designed to detect changes in outcomes related to cardiac events. In addition, generic instruments have been shown to be less responsive to changes after treatment than are disease-specific instruments [12].

Several disease-specific HRQOL questionnaires, such as the Seattle Angina Questionnaire (SAQ) and the Angina Pectoris Quality of Life Questionnaire (APQLQ), can assess the HRQOL of patients with coronary artery disease. The SAQ has been translated into the Korean language [13] and is used to evaluate outcomes related to coronary artery disease [14]. Unfortunately, the SAQ does not include items related to the adverse effects of CABG or PCI. Thus, it is difficult to measure changes after CABG or PCI, such as adverse effects.

The Coronary Revascularization Outcome Questionnaire (CROQ) was developed to assess not only the HRQOL of patients with coronary artery disease, but also the adverse effects of CABG or percutaneous transluminal coronary angiography (PTCA) [15]. The validity and reliability of the CROQ- CABG and PTCA have been well established [15], and this inventory is just as responsive as the SAQ and even more responsive to change than the generic questionnaire [12]. It has also been translated into Japanese and Persian languages [16, 17], but a Korean version is not available. In Korea, PCI is more commonly used than CABG to treat IHD [18]. In this light, the aim of this study was to (1) translate the CORQ-PTCA into Korean and (2) assess the reliability and validity of the CORQ-PTCA among Korean-speaking patients.

## Methods

### CROQ-PTCA

The CRQO-PTCA has two versions: the CROQ-PTCA Pre-revascularization and the CROQ-PTCA Post-revascularization. Both versions contain 32 core evaluation items: symptoms (7 items), physical functioning (8 items), cognitive functioning (3 items), and psychosocial functioning (14 items), as well as one descriptive item that is not included in the scale scores [15]. The CROQ has additional post-revascularization items about satisfaction (6 items) and adverse effects (6 items) and two descriptive items that are not included in the scale scores. Each item is rated using three six-point Likert scales. The items in each scale are summed and then converted to a 0–100 scale, with 100 representing the best outcome.

#### Translation

The CROQ-PTCA was translated using standardized forward-backward procedures [19]. First, the original version of the CROQ-PTCA was obtained from the original author, and permission was granted for its use in this study. Two nursing faculty members who were native Korean speakers and proficient in English translated it into Korean. The translated version was evaluated for clarity, word choice, and agreement with the original version by an expert panel, including a cardiologist, a preventive medicine physician, and two nursing faculty members. The expert panel made the following changes: (1) the terms “half a mile” and “100 yards” were changed to “around 1 km” and “around 100 m” because Korea uses the metric system. (2) The word “groin” was changed to “puncture site” because both the radial and femoral sites are used for catheter insertion in the PCI procedure in Korea. After the expert’s evaluation, the tool was back-translated into English by a bilingual individual. The expert panel then re-evaluated the back-translated version of the CROQ-PTCA to assess its agreement with the original version. After a professional Korean editor proofread the final version, a pilot study with 10 patients was conducted to evaluate whether the Korean version of the CROQ-PTCA was clear and easy to understand. This investigation led to some minor modifications of the Korean medical terms.

### Patients

Participants were recruited from cardiologic clinics in a university hospital in J City, Korea. The inclusion criteria were: (1) age 18 years or older; (2) diagnosed with IHD by a cardiologist; (4) ability to read and write Korean; and (5) intact cognitive function. The exclusion criterion was (1) previous experiences with PCI or CABG. Eligibility to participate was confirmed by the cardiologist who was one of the co-investigators in this study. The eligible patients were sorted into two groups on the basis of treatment stage: a pre-PCI group who were recommended to undergo elective PCI by a cardiologist and a post-PCI group who had undergone PCI via the radial or femoral artery three months prior. The three-month point was selected because the majority of patients should have recovered from the procedure by then, and only a few patients would still be experiencing adverse effects.

Patients who met the inclusion criteria were invited to participate in the study. After providing written informed consent, the patients were asked to complete a series of questionnaires including the CROQ-PTCA, SAQ-K, and SF-12-K with demographic characteristics. A total of 209 patients participated in the pre-PCI assessment. In addition, 169 patients participated in the post-PCI assessment. Twenty-eight patients completed both pre- and post-PCI assessments. The data were collected between January 2014 and December 2014, after receiving approval from the institutional review board.

### Measures

The Seattle Angina Questionnaire-Korean (SAQ-K) was used to assess the concurrent validity of the CROQ-PTCA (K). The SAQ-K consists of five domains: physical limitations (nine items), angina stability (one item), angina frequency (two items), treatment satisfaction (four items), and quality of life (three items) [20]. Each item is rated using 5–6-point Likert scales. Items in each scale are summed and then converted to a 0–100 scale. A score of 100 points represents the best outcome.

The SF-12 was also used to assess the concurrent validity of the CROQ-PTCA (K). The SF-12 is an abbreviated version of the SF-36 and contains 12 items covering eight domains (physical functioning, role-physical, bodily pain, general health, vitality, social functioning, role-emotional, and mental health), which produce two summary scores: the physical component summary (PCS) and the mental component summary (MCS) scores. The PCS and the MCS are standardized (mean = 50, SD = 10). For each of the eight domains, the items are summed and then converted to a 0–100 scale, with high scores indicating better outcomes.

### Statistical analysis

Patient characteristics in the pre-PCI and post-PCI groups were analyzed using descriptive statistics. Scores for each domain of the CROQ-PTCA were evaluated by descriptive analysis. Cronbach’s alpha was used to examine the internal consistency, and the item-total correlation coefficients were also computed. A Cronbach’s alpha $\ge 0$.70 and item-total correlation coefficients >0.2 were deemed statistically acceptable [21]. Spearman’s rank correlation coefficients between the scores of the CROQ-PTCA (K), SAQ-K, and SF-12 (K) questionnaires were used to assess the concurrent validity. To evaluate the construct validity, an exploratory factor analysis (EFA) with varimax rotation was performed. After an assessment of all eigenvalues greater than 1, the number of domains was determined for the core items, those associated with treatment satisfaction, and items related to the adverse effects of PCI. Responsiveness was the ability of the scales to detect clinically important changes over time [22]. The Wilcoxon signed-rank test was used to compare the results before and three months after revascularization. Effect size statistics were calculated as the mean change score between pre- and post-PCI assessments divided by the standard deviation of scores at the pre-PCI assessment [22].

## Results

### Patient characteristics

Table 1 shows the characteristics of participants. Most participants were male (80.9% in the pre-PCI group, 76.9% in the post-PCI group) and lived with a spouse (79.7% in the pre-PCI group, 74.0% in the post-PCI group). The mean age was 60.8 (±10.56) years in the pre-PCI group and 61.6 (±9.76) years in the post-PCI group. The average number of years that patients had been receiving treatment for ischemic heart disease was 3.51 in the pre-PCI group and 2.72 in the post-PCI group.

**Table 1:** Patient Characteristics

Variables		Pre-PCI (*n* = 209)		Post-PCI (*n* = 169)	
		*n*	%	*n*	%
Sex	Male	169	80.9	130	76.9
	Female	40	19.1	39	23.1
Education	≤ Middle school	97	46.4	94	55.6
	≥ High school	112	53.6	75	44.4
Income	≤100	64	30.9	58	34.3
(10,000 won)	101–300	94	45.4	82	48.5
	≥301	49	23.7	29	17.2
Living arrangement	With spouse	165	79.7	125	74.0
	With someone	9	4.3	19	11.2
	Alone	33	15.9	25	14.8
Employed	No	70	33.5	65	38.5
	Yes	139	66.5	104	61.5
Puncture site	Radial artery			85	50.3
	Femoral artery			84	49.7
		Mean	SD	Mean	SD
Age (years)		60.84	10.56	61.64	9.76
Duration of treatment (years)		3.51	4.79	2.72	2.90

*PCI* percutaneous coronary intervention

### Scores and Reliability of the CROQ-PTCA (K)

The mean score of each domain and the reliability of the CROQ-PTCA (K) are shown in Table 2. Cronbach’s alphas were computed for each domain based on the pre and post-PCI data as indicators of internal consistency. Cronbach’s alphas of the domains of symptoms, physical functioning, cognitive functioning, and psychosocial functioning in the core items in both the pre- and post-PCI assessments as well as the domain of satisfaction in the post-PCI assessment were ≥0.70. However, the domain of adverse effects had a Cronbach’s alpha of 0.47. The means of the item-total correlations for the domains of the CROQ-PTCA (K) ranged from 0.27–0.83.

**Table 2:** Scores and Reliability of the CROQ-PTCA (K)

	Mean (SD)	Reliability (Cronbach’s α)	Item-total correlation (Mean)
CROQ-PTCA Pre (*N* = 209)			
Symptoms	74.88 (21.46)	0.80	0.16–0.77 (0.52)
Physical functioning	92.14 (15.95)	0.88	0.49–0.79 (0.68)
Cognitive functioning	96.91 (10.90)	0.91	0.75–0.87 (0.83)
Psychosocial functioning	93.22 (15.05)	0.96	0.65–0.88 (0.79)
CROQ-PTCA Post (*N* = 169)			
Symptoms	94.76 (10.35)	0.78	0.37–0.71 (0.52)
Physical functioning	96.93 (9.69)	0.89	0.64–0.78 (0.70)
Cognitive functioning	96.21 (8.78)	0.89	0.84–0.89 (0.83)
Psychosocial functioning	85.52 (13.03)	0.92	0.42–0.77 (0.64)
Satisfaction	85.83 (14.66)	0.82	0.51–0.69 (0.61)
Adverse effects	98.35 (5.44)	0.47	0.08–0.45 (0.27)
Radial artery (*n* = 85)	98.62 (4.82)	0.33	0.03–0.51 (0.22)
Femoral artery (*n* = 84)	98.06 (6.01)	0.59	0.08–0.72 (0.42)

*CROQ* Coronary Revascularization Outcome Questionnaire, *PTCA* percutaneous transluminal coronary angioplasty

### Validity

The results of the exploratory factor analyses (EFA) of the 32 core items of the CROQ-PTCA (K) are shown in Table 3. The analyses yielded seven factors that explained a total of 79.4% of the variance in the pre-PCI test and 75.7% in the post-PCI test. The KMO measure was 0.89 on the pre-PCI test and 0.84 on the post-PCI test. Table 4 shows the results of the EFA of items regarding satisfaction with and adverse effects of the CROQ-PTCA (K)-Post. The domain of satisfaction was identified as having two factors, which explained 80.3% of the variance. Regarding the items related to these adverse effects, three factors were identified, which explained 76.9% of the variance.

**Table 3:** Factor analyses of CROQ-PTCA (K) Core Items in the Pre- and Post-PCI tests

	Pre-PCI							Post-PCI						
	Factor 1	Factor 2	Factor 3	Factor 4	Factor 5	Factor 6	Factor 7	Factor 1	Factor 2	Factor 3	Factor 4	Factor 5	Factor 6	Factor 7
Symptoms														
Chest pain	0.07	0.03	-0.05	0.02	0.91	0.11	0.09	0.18	-0.02	0.16	0.01	-0.12	0.75	0.38
Chest discomfort	0.03	0.08	-0.05	0.04	0.90	0.11	0.04	0.14	-0.11	0.23	-0.04	0.01	0.69	0.33
SOB	0.09	0.13	0.23	0.28	0.63	0.00	-0.33	0.61	0.12	0.12	-0.09	0.33	0.30	-0.08
Radiating pain	0.10	0.06	0.15	0.11	0.55	-0.01	0.55	0.35	0.26	0.03	0.18	0.07	0.20	0.69
Palpitations	0.23	-0.13	0.32	0.02	0.20	0.52	0.01	0.16	0.20	0.12	0.16	0.13	0.13	0.66
Frequency of use of nitroglycerin	0.06	0.32	0.46	0.25	0.05	-0.29	-0.08	0.03	0.12	-0.07	0.00	0.10	0.78	-0.14
Global trouble	0.24	0.09	0.22	0.41	0.69	0.04	0.03	0.53	-0.01	0.27	-0.11	-0.08	0.52	0.28
Physical Functioning														
Moderate activities	0.28	0.23	0.17	0.45	0.09	0.66	-0.18	0.66	0.14	0.00	0.16	0.08	0.16	0.38
Lifting and carrying	0.34	0.34	0.04	0.35	0.19	0.56	-0.28	0.80	0.12	0.15	0.05	-0.11	0.05	0.14
Climbing flights of stairs	0.30	0.09	0.18	0.72	0.25	0.10	-0.05	0.62	0.25	0.02	0.15	0.11	0.38	-0.21
Climbing one flight of stairs	0.15	0.15	0.27	0.76	0.15	0.22	-0.01	0.78	0.17	0.01	0.19	0.17	0.09	-0.02
Bending, kneeling, stooping	0.11	0.40	0.09	0.46	-0.03	0.35	0.20	0.77	-0.04	0.06	0.10	0.17	-0.06	0.26
Walking about 1 km	0.33	0.20	0.13	0.76	0.09	0.04	0.11	0.68	0.26	0.06	0.12	-0.04	0.31	-0.21
Walking about 100 m	0.21	0.21	0.20	0.76	0.16	0.16	0.25	0.83	0.12	0.00	0.14	-0.07	0.02	0.09
Bathing or dressing	0.08	0.20	0.26	0.18	0.05	0.71	0.26	0.82	-0.11	0.07	0.00	-0.01	-0.14	0.24
Cognitive Functioning														
Difficulty reasoning	0.18	0.53	0.51	0.26	-0.01	0.20	0.41	0.09	0.22	0.18	0.32	0.85	0.03	0.08
Forgetting things	0.14	0.35	0.49	0.34	-0.02	0.05	0.45	0.01	0.07	0.18	0.25	0.84	-0.03	0.02
Difficulty with activities involving concentration	0.25	0.49	0.44	0.27	0.01	0.13	0.47	0.08	0.25	0.14	0.30	0.82	0.04	0.11
Psychosocial Functioning														
Worries about heart condition	0.87	0.24	0.21	0.21	0.11	0.13	0.07	0.04	0.17	0.88	0.05	0.12	0.09	0.00
Worries about doing too much	0.83	0.22	0.23	0.29	0.12	0.14	0.02	0.06	0.26	0.87	0.05	0.19	0.09	0.05
Worries about having a heart attack or dying	0.85	0.25	0.24	0.26	0.11	0.11	0.06	0.11	0.25	0.91	0.03	0.10	0.08	0.08
Fear of pain or discomfort	0.84	0.30	0.16	0.19	0.10	0.13	0.05	0.10	0.25	0.91	0.03	0.05	0.09	0.08
Uncertain about the future	0.49	0.61	0.34	0.24	0.06	0.23	0.02	0.07	0.48	0.64	0.19	0.14	-0.04	0.02
Depressed	0.21	0.88	0.18	0.10	0.14	-0.03	-0.01	0.07	0.84	0.20	0.12	0.11	0.02	-0.04
Frustrated or impatient	0.27	0.87	0.15	0.20	0.12	0.04	-0.01	0.18	0.87	0.18	-0.01	0.13	0.09	0.04
Interferences with enjoyment of life	0.58	0.56	0.23	0.23	0.06	0.25	0.10	0.19	0.75	0.36	0.18	0.06	0.01	0.16
Difficult to keep a positive outlook	0.48	0.65	0.29	0.15	0.06	0.25	0.13	0.12	0.85	0.23	0.15	0.15	-0.02	0.20
Difficult to plan ahead	0.39	0.66	0.36	0.19	0.08	0.21	0.22	0.08	0.78	0.33	0.16	0.16	0.03	0.16
Overprotective family or friends	0.20	0.17	0.88	0.14	0.07	0.15	0.05	0.06	0.06	-0.03	0.78	0.07	0.02	0.10
Feeling like a burden	0.26	0.16	0.87	0.14	0.08	0.18	0.07	0.11	0.18	0.06	0.73	0.44	-0.04	0.09
Feeling restricted in social activities	0.21	0.20	0.86	0.15	0.07	0.22	0.09	0.16	0.10	0.13	0.80	0.23	0.00	0.00
Worries about going too far from home	0.27	0.36	0.67	0.33	0.07	0.27	0.06	0.22	0.21	0.13	0.72	0.30	-0.04	0.15
Contribution rate			79.4%								75.7			
Kaiser-Meyer-Olkin			0.89								0.84			

*CROQ* Coronary Revascularization Outcome Questionnaire, *PTCA* percutaneous transluminal coronary angioplasty, *PCI* percutaneous coronary intervention, *SOB* shortness of breath

**Table 4:** Factor Analyses of CROQ-PTCA (K) Post Items

Satisfaction (*n* = 169)			Adverse effects (*n* = 169)			
	Factor 1	Factor 1		Factor 1	Factor 1	Factor 1
Satisfaction with the results of the operation	0.89	0.26	Pain at the puncture site	<0.01	0.84	0.24
Satisfaction with information about the operation	0.96	0.18	Tenderness around the puncture site	0.17	0.19	0.71
Satisfaction with information about recovering from the operation	0.96	0.16	Numbness or tingling at the puncture site	-0.02	0.89	-0.05
Heart condition compared to before the operation	0.17	0.80	Bruising around the puncture site	0.94	-0.02	0.02
Speed of recovery	0.13	0.81	Catheter-related problems at the puncture site	-0.07	-0.03	0.85
Expectation of results	0.20	0.80	Concern over the appearance of bruises	0.94	0.01	0.07
Contribution rate	80.3%		Contribution rate	76.9%		
Kaiser-Meyer-Olkin	0.76		Kaiser-Meyer-Olkin	0.52		

*CROQ* Coronary Revascularization Outcome Questionnaire, *PTCA* percutaneous transluminal coronary angioplasty

Table 5 shows the correlation among the scores of the CROQ-PTCA, SAQ, and SF-12. The CROQ symptom had positive correlations with the PCS of the SF-12 in the post-test and the angina frequency scale of the SAQ in the pre- (r = 0.72) and post-PCI (r = 0.79) tests. The CROQ physical functioning was positively correlated with the PCS of the SF-12 (r = 0.62 and r = 0.43) and the physical limitation scale of the SAQ (r = 0.86 and r = 0.87). The CROQ cognitive functioning was positively correlated with PCS (r = 0.50 and r = 0.37) and MCS (r = 0.34 and r = 0.43) of the SF-12 for both tests and with the quality of life scale of the SAQ in the pre-PCI test (r = 0.28). The CROQ psychosocial functioning was positively correlated with the PCS (r = 0.57 and r = 0.49) and MCS (r = 0.31 and r = 0.52) of the SF-12 and the quality of life scale of the SAQ (r = 0.45 and r = 0.45) on both tests. The CROQ satisfaction was positively correlated with the treatment satisfaction scale of the SAQ (r = 0.59). The CROQ’s adverse effects were positively correlated with the quality of life scale of the SAQ (r = 0.22).

**Table 5:** Concurrent Validity of the CROQ-PTCA (K)

CROQ scale	SF-12		SAQ				
	PCS (*N* = 209)	MCS (*N* = 209)	Physical Limitation Scale (*N* = 208)	Angina Stability Scale (*N* = 209)	Angina Frequency Scale (*N* = 209)	Treatment Satisfaction Scale (*N* = 209)	Quality of Life Scale (*N* = 209)
CROQ-PTCA Pre							
Symptoms	0.13	-0.21**	0.54**	-0.17*	0.72**	0.44**	0.47**
Physical functioning	0.62**	0.23**	0.86**	0.38**	0.61**	0.18*	0.40**
Cognitive functioning	0.50**	0.34**	0.52**	0.22**	0.47**	0.09	0.28**
Psychosocial functioning	0.57**	0.31**	0.70**	0.28**	0.56**	0.23**	0.45**
	PCS (*N* = 169)	MCS (*N* = 169)	The Physical Limitation Scale (*N* = 160)	The Angina Stability Scale (*N* = 169)	The Angina Frequency Scale (*N* = 169)	The Treatment Satisfaction Scale (*N* = 169)	The Quality of Life scale (*N* = 169)
CROQ-PTCA Post							
Symptoms	0.27**	0.08	0.57**	0.02	0.79**	0.17*	0.39**
Physical functioning	0.43**	0.22**	0.87**	0.28**	0.55**	0.16*	0.27**
Cognitive functioning	0.37**	0.43**	0.10	0.02	<0.01	0.14	0.02
Psychosocial functioning	0.49**	0.52**	0.33**	0.05	0.18*	0.19*	0.45**
Satisfaction	0.36**	0.32**	0.15	0.01	0.07	0.591*	0.19*
Adverse effects	0.07	-0.06	0.14	-0.14	0.06	0.15	0.22**

*p < 0.05 **p < 0.01

*CROQ* Coronary Revascularization Outcome Questionnaire, *PTCA* percutaneous transluminal coronary angioplasty, *SAQ* Seattle Angina Questionnaire, *PCS* physical component summary, *MCS* mental component summary

### Responsiveness

Table 6 shows the results of the responsiveness analysis for the CROQ based on the data of 28 patients who completed both the pre- and post-PTCA questionnaires. Large effect sizes were observed for the symptoms and the psychosocial functioning scales.

**Table 6:** Responsiveness of the CROQ-PTCA (K)

CROQ scale	Mean (SD)			Effect size
	Pre Mean (median)	Post Mean (median)	Change Mean (median)	
CROQ-PTCA (*N* = 28)				
Symptoms	77.83 (82.1)	94.13 (100.0)	16.31 (17.9)**	1.30
Physical functioning	91.96 (100.0)	94.20 (100.0)	2.23 (0.0)	0.12
Cognitive functioning	95.00 (100.0)	97.62 (100.0)	2.62 (0.0)	0.41
Psychosocial functioning	94.13 (100.0)	85.84 (92.0)	-8.29 (-8.0)**	-0.50

**p < 0.01

*CROQ* Coronary Revascularization Outcome Questionnaire, *PTCA* percutaneous transluminal coronary angioplasty

## Discussion

To our knowledge, this is the first report on the translation and validation of the CROQ-PTCA in the Korean language. The results indicated that the Korean version of the CROQ-PTCA was psychometrically sound and appropriate for patients who undergo PCI procedures.

Regarding the translation procedure, the changes in the terms used are consistent with the cultural and procedural characteristics in Korea. In this study, about half of the patients received PCI via a radial artery. Evaluation by the expert group and a pilot test confirmed the content validity of the CROQ-PTCA (K).

According to the results of the analysis for the Cronbach’s alpha of each domain of the CROQ-PTCA (K), all domains in the CROQ core items and the CROQ satisfaction met the criteria value, which was a Cronbach’s alpha of 0.7[21]. All items showed higher than 0.20 item-total correlation coefficients in the CROQ core items [21], except ‘frequency of use of nitroglycerin’ (0.16) in the CROQ symptom in the pre-PCI assessment. The low item-total correlation of ‘frequency of use of nitroglycerin’ might be associated with the fact that only 18 patients had used nitroglycerin (data not presented in tables) among 209 participants in the pre-test. However, the CROQ adverse effects showed a low Cronbach’s alpha of 0.47 and a mean of the item-total correlation coefficients of 0.27. Adverse effects described in the original CROQ-PTCA were those that occur when a femoral artery was used, and the Cronbach’s alpha for this original version was 0.87 [15]. In a Japanese study, the Cronbach’s alpha of the adverse effects was 0.83, but no information about puncture site was available [16]. However, many studies have reported that the radial approach is a safe method compared to the femoral approach due to a decreased risk of puncture site complications [23–25]. In our study, the radial artery was used for half of the PCI procedures (Cronbach’s α = 0.33), and the mean score of adverse effects was very high (98.35 of 100), indicating almost no puncture site complications. Thus, the CROQ adverse effects may not be adequate for patients receiving PCI via the radial artery.

Seven factors were identified in the results of the EFA of the CROQ-PTCA (K) core items, with 79.4% and 75.7% of the contribution ratios. All items had a loading factor greater than 0.4 for the respective domain. The original version has four domains, but has not been the subject of EFA [15]. In the Japanese study, the four identified factors were almost the same as those found in the original version, but the contribution ratio was only 54.3% [16]. In our study, CROQ psychosocial functioning was separated into three factors of social activities, feelings about oneself, and feelings about one’s relationships with others. Physical functioning had two factors in the pre-test but only one factor on the post-test. Some items of the symptoms were identified as the same factor of physical functioning, such as palpitations on the pre-test and shortness of breathing on the post-test. The CROQ cognitive functioning contained one factor for both tests, while three items were included for psychosocial functioning in the pre-test. Although our findings were different from those of a previous study [16], the factors of the CROQ-PTCA (K) were reasonably identified.

In the results of the EFA of the CROQ-PTCA Post items, all items had a greater than 0.4 loading factor. The CROQ satisfaction was separated into two factors. Three items about satisfaction were identified as one factor, while three items about expectation were grouped into another factor. The CROQ adverse effects were separated into three factors of bruising, pain and numbness, and tenderness and other problems. In the Japanese study of 42 patients, however, one factor was identified in each domain of the CROQ-PTCA Post items [16]. As previously mentioned, there was no information about the puncture site of 42 patients in the Japanese study. In our study, participants reported almost no puncture site complications. Therefore, additional studies in patients undergoing PCI via the femoral artery or who experience more severe adverse effects are needed. In addition, further research using earlier assessment points than three months will be required to assess the adverse effects.

The concurrent validity of the CROQ-PTCA (K) was supported by the pattern of association among the CROQ, SF-12, and SAQ. The CROQ symptoms were significantly correlated with the PCS of SF-12 in the post-test and with the angina frequency scale of the SAQ. The CROQ physical functioning was significantly correlated with the PCS of the SF-12 and the physical limitation scale of the SAQ. The CROQ cognitive functioning and psychosocial functioning were significantly correlated with the MCS of the SF-12. The CROQ satisfaction was also significantly correlated with the treatment satisfaction scale of the SAQ. All domains of the CROQ-PTCA (K), except the cognitive functioning domain on the post-PCI test, were significantly correlated with the quality of life domain of the SAQ. As a result, the concurrent validity of the CROQ-PTCA (K) was confirmed.

In this study, the responsiveness of the CROQ-PTCA (K) was verified. The domain of symptoms was significantly improved after PCI. However, psychosocial functioning significantly declined after PCI, while the domains of physical and cognitive functioning did not significantly change. This finding can be explained by the characteristics of our participants. Most participants in this study were recruited from the outpatient department and were therefore not severely ill. We included only patients who had undergone elective PCI instead of an urgent procedure. Before the cardiac event, these patients likely considered themselves to be healthy, so their physical, cognitive, and psychosocial functioning domains on the pre-PCI test showed high scores. However, after the PCI, they might have realized the severity of their disease and more severely perceived the impact of the disease on their lives. Given its demonstrated good responsiveness, the CROQ-PTCA (K) may be appropriate to detect important differences in outcomes before and after PCI.

Our study has several limitations that should be mentioned. First, the investigation was restricted to patients receiving elective PCI in hospitals in Korea. Further validation research with patients undergoing not only elective, but also urgent PCI is needed. Secondly, the CROQ’s adverse effects showed low reliability. It is possible that the results would have differed if we had used earlier assessment points than three months to assess the associated adverse effects. Thirdly, the sample size used to evaluate the responsiveness of the CROQ was quite small. Further research will be needed to investigate the responsiveness of the CROQ for changes in a large sample.

## Conclusions

The CROQ was originally developed to assess patients’ specific health-related quality of life (HRQOL) and also to evaluate treatment effectiveness from the patient’s perspective. The Korean version of the CROQ-PTCA demonstrated good psychometric properties and was responsive to change. Thus, the CROQ-PTCA (K) is appropriate and acceptable for detecting meaningful changes that take place before and after PCI.

## Abbreviations

APQLQ: Angina Pectoris Quality of Life Questionnaire
CABG: coronary artery bypass graft surgery
CROQ: Coronary Revascularization Outcome Questionnaire
EFA: exploratory factor analysis
HRQOL: health-related quality of life
IHD: ischemic heart disease
MCS: mental component summary
NHD: Nottingham Health Profile
PCI: percutaneous coronary intervention
PCS: physical component summary
PTCA: percutaneous transluminal coronary angioplasty
SAQ: Seattle Angina Questionnaire
SOB: shortness of breath
